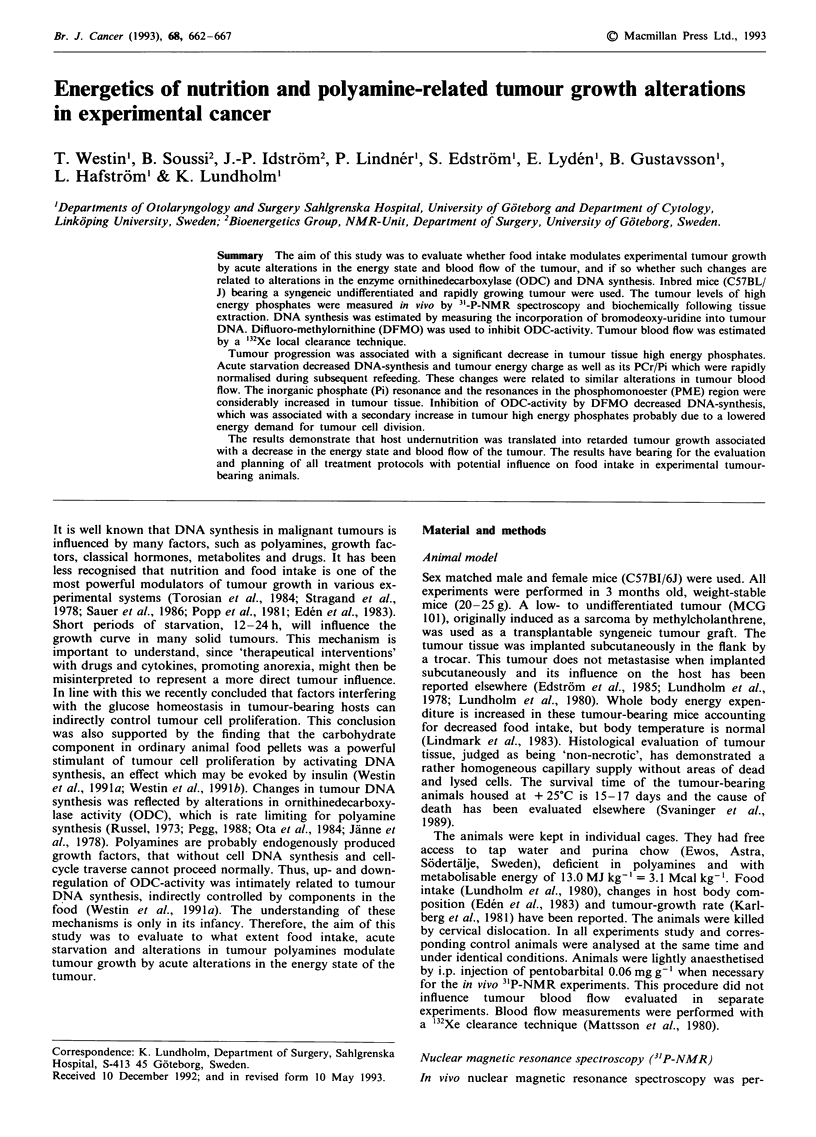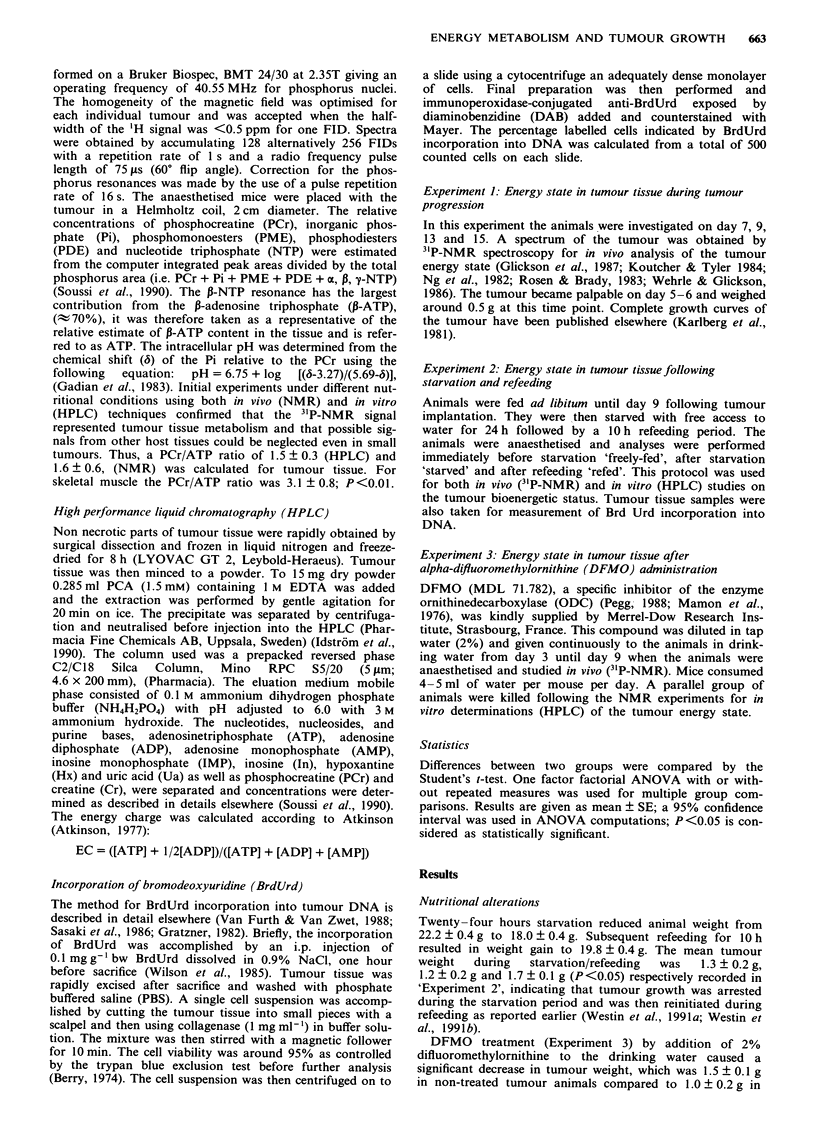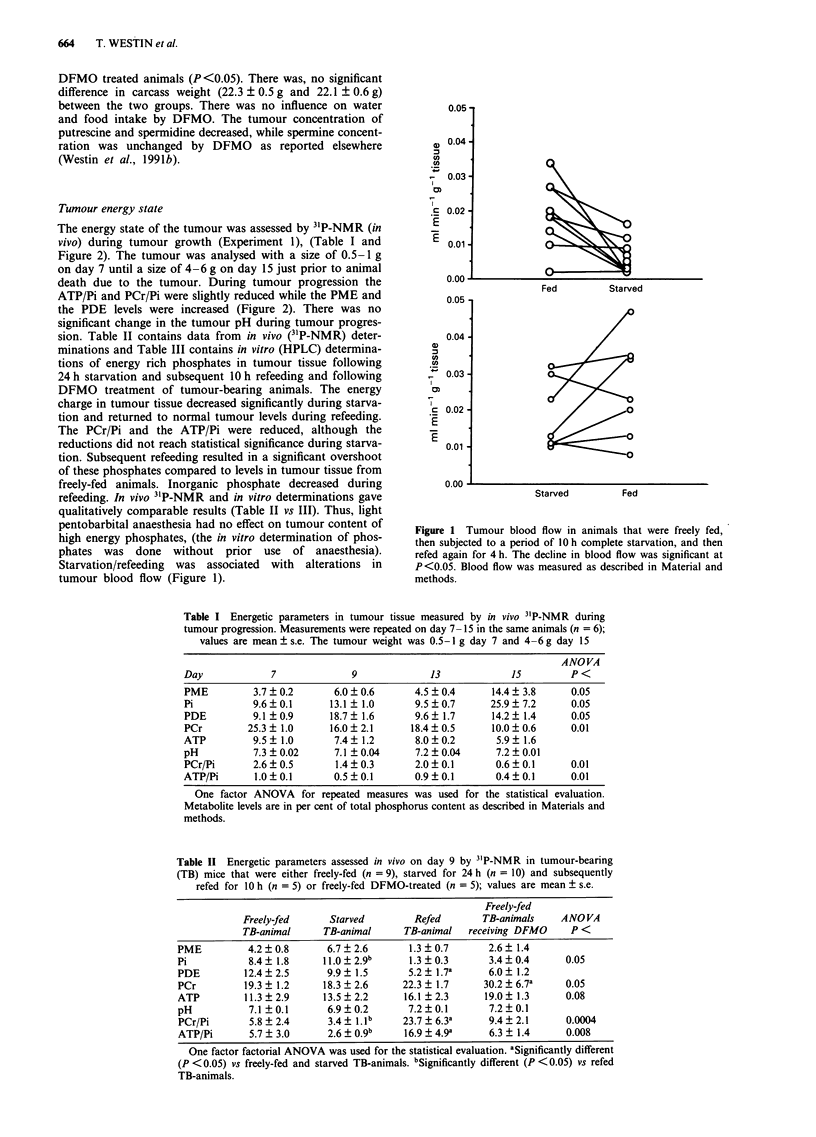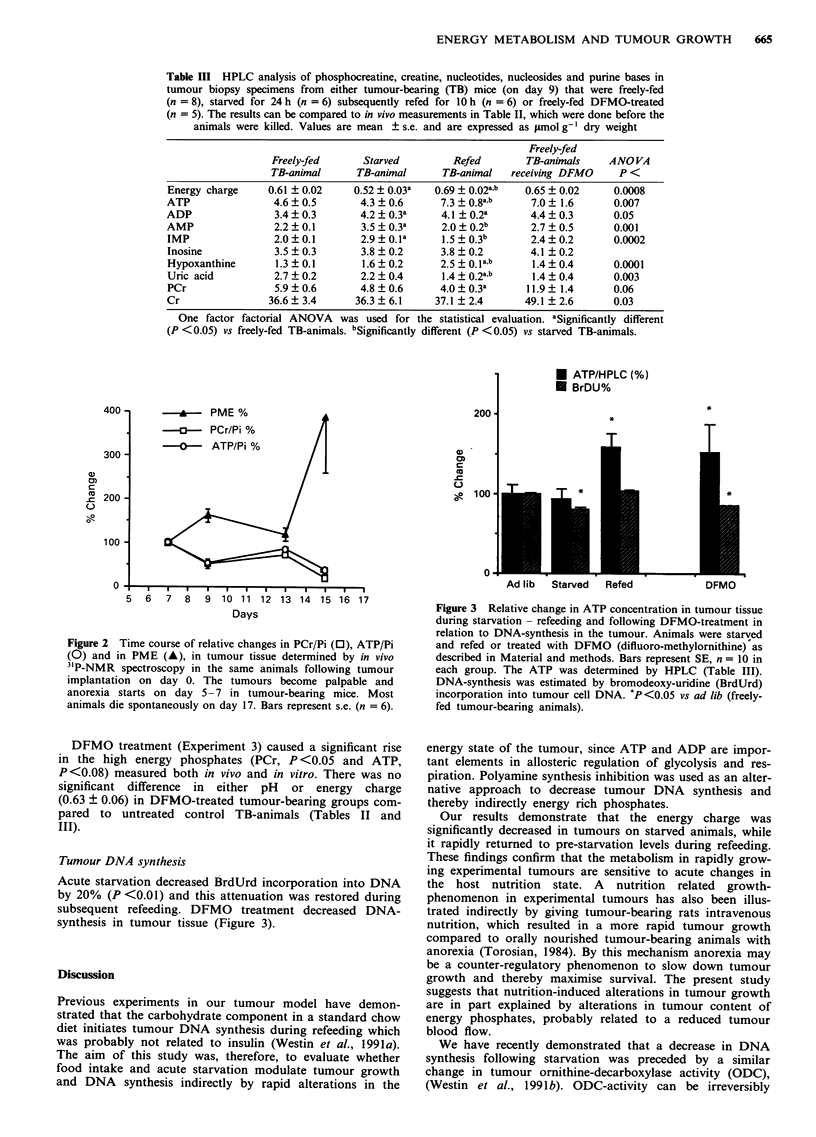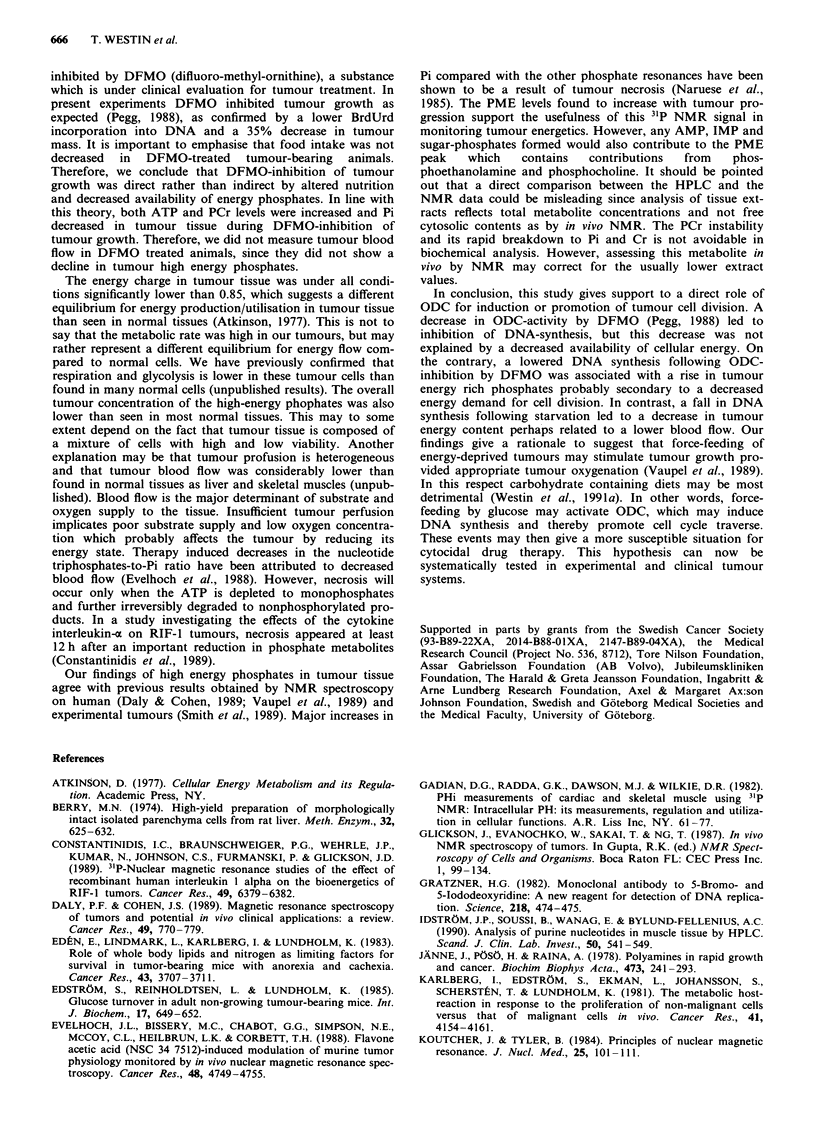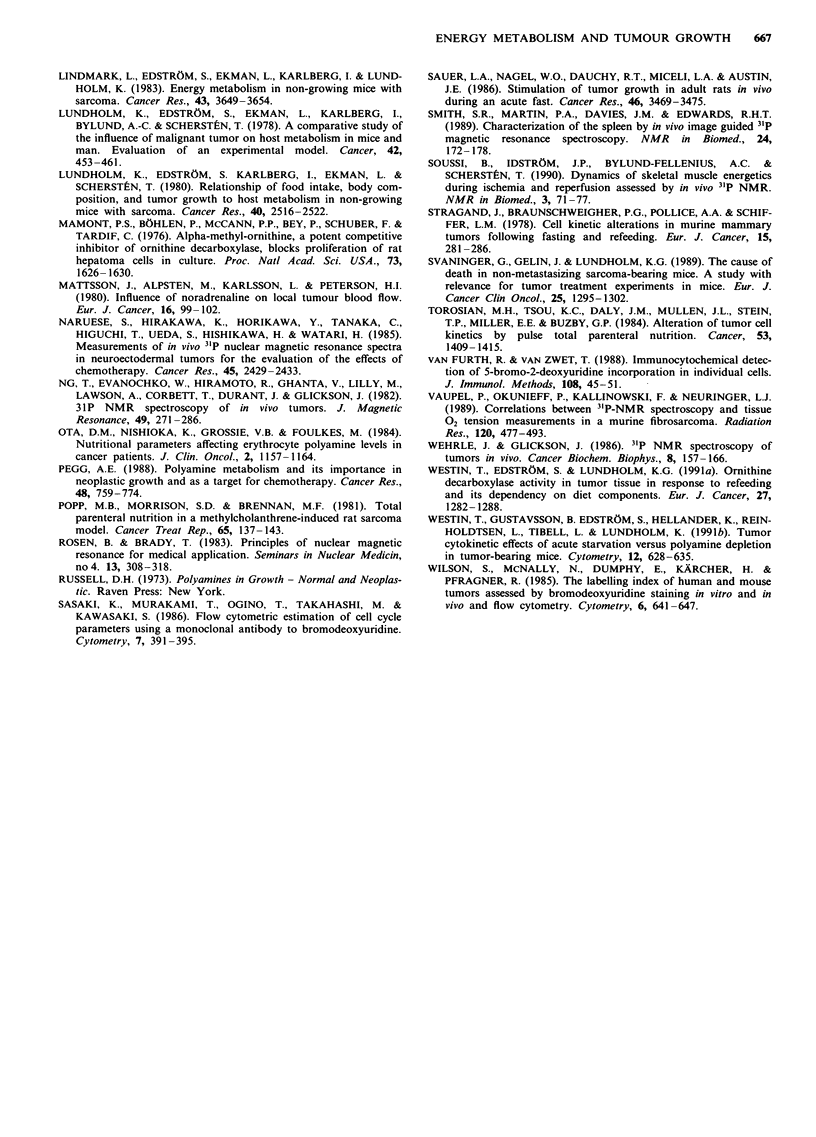# Energetics of nutrition and polyamine-related tumor growth alterations in experimental cancer.

**DOI:** 10.1038/bjc.1993.405

**Published:** 1993-10

**Authors:** T. Westin, B. Soussi, J. P. Idström, P. Lindnér, S. Edström, E. Lydén, B. Gustavsson, L. Hafström, K. Lundholm

**Affiliations:** Department of Otolaryngology, Sahlgrenska Hospital, University of Göteborg, Sweden.

## Abstract

The aim of this study was to evaluate whether food intake modulates experimental tumour growth by acute alterations in the energy state and blood flow of the tumour, and if so whether such changes are related to alterations in the enzyme ornithinedecarboxylase (ODC) and DNA synthesis. Inbred mice (C57BL/J) bearing a syngeneic undifferentiated and rapidly growing tumour were used. The tumour levels of high energy phosphates were measured in vivo by 31-P-NMR spectroscopy and biochemically following tissue extraction. DNA synthesis was estimated by measuring the incorporation of bromodeoxy-uridine into tumour DNA. Difluoro-methylornithine (DFMO) was used to inhibit ODC-activity. Tumour blood flow was estimated by a 132Xe local clearance technique. Tumour progression was associated with a significant decrease in tumour tissue high energy phosphates. Acute starvation decreased DNA-synthesis and tumour energy charge as well as its PCr/Pi which were rapidly normalised during subsequent refeeding. These changes were related to similar alterations in tumour blood flow. The inorganic phosphate (Pi) resonance and the resonances in the phosphomonoester (PME) region were considerably increased in tumour tissue. Inhibition of ODC-activity by DFMO decreased DNA-synthesis, which was associated with a secondary increase in tumour high energy phosphates probably due to a lowered energy demand for tumour cell division. The results demonstrate that host undernutrition was translated into retarded tumour growth associated with a decrease in the energy state and blood flow of the tumour. The results have bearing for the evaluation and planning of all treatment protocols with potential influence on food intake in experimental tumour-bearing animals.


*Br. J. Cancer* (1993), **68**, 662–667 © Macmillan Press Ltd., 1993

### Energetics of nutrition and polyamine-related tumour growth alterations in experimental cancer

T. Westin[1], B. Soussi[2], J.-P. Idström[2], P. Lindnér[1], S. Edström[1], E. Lydén[1], B. Gustavsson[1], L. Hafström[1] & K. Lundholm[1]

[1]*Departments of Otolaryngology and Surgery Sahlgrenska Hospital, University of Göteborg and Department of Cytology, Linköping University, Sweden;* [2]*Bioenergetics Group, NMR-Unit, Department of Surgery, University of Göteborg, Sweden.*

**Summary** The aim of this study was to evaluate whether food intake modulates experimental tumour growth by acute alterations in the energy state and blood flow of the tumour, and if so whether such changes are related to alterations in the enzyme ornithinedecarboxylase (ODC) and DNA synthesis. Inbred mice (C57BL/J) bearing a syngeneic undifferentiated and rapidly growing tumour were used. The tumour levels of high energy phosphates were measured *in vivo* by $^{31}$-P-NMR spectroscopy and biochemically following tissue extraction. DNA synthesis was estimated by measuring the incorporation of bromodeoxy-uridine into tumour DNA. Difluoro-methylornithine (DFMO) was used to inhibit ODC-activity. Tumour blood flow was estimated by a $^{132}$Xe local clearance technique.

Tumour progression was associated with a significant decrease in tumour tissue high energy phosphates. Acute starvation decreased DNA-synthesis and tumour energy charge as well as its PCr/Pi which were rapidly normalised during subsequent refeeding. These changes were related to similar alterations in tumour blood flow. The inorganic phosphate (Pi) resonance and the resonances in the phosphomonoester (PME) region were considerably increased in tumour tissue. Inhibition of ODC-activity by DFMO decreased DNA-synthesis, which was associated with a secondary increase in tumour high energy phosphates probably due to a lowered energy demand for tumour cell division.

The results demonstrate that host undernutrition was translated into retarded tumour growth associated with a decrease in the energy state and blood flow of the tumour. The results have bearing for the evaluation and planning of all treatment protocols with potential influence on food intake in experimental tumour-bearing animals.

It is well known that DNA synthesis in malignant tumours is influenced by many factors, such as polyamines, growth factors, classical hormones, metabolites and drugs. It has been less recognised that nutrition and food intake is one of the most powerful modulators of tumour growth in various experimental systems (Torosian *et al.*, 1984; Stragand *et al.*, 1978; Sauer *et al.*, 1986; Popp *et al.*, 1981; Edén *et al.*, 1983). Short periods of starvation, 12–24 h, will influence the growth curve in many solid tumours. This mechanism is important to understand, since 'therapeutical interventions' with drugs and cytokines, promoting anorexia, might then be misinterpreted to represent a more direct tumour influence. In line with this we recently concluded that factors interfering with the glucose homeostasis in tumour-bearing hosts can indirectly control tumour cell proliferation. This conclusion was also supported by the finding that the carbohydrate component in ordinary animal food pellets was a powerful stimulant of tumour cell proliferation by activating DNA synthesis, an effect which may be evoked by insulin (Westin *et al.*, 1991*a*; Westin *et al.*, 1991*b*). Changes in tumour DNA synthesis was reflected by alterations in ornithinedecarboxylase activity (ODC), which is rate limiting for polyamine synthesis (Russel, 1973; Pegg, 1988; Ota *et al.*, 1984; Jänne *et al.*, 1978). Polyamines are probably endogenously produced growth factors, that without cell DNA synthesis and cell-cycle traverse cannot proceed normally. Thus, up- and down-regulation of ODC-activity was intimately related to tumour DNA synthesis, indirectly controlled by components in the food (Westin *et al.*, 1991*a*). The understanding of these mechanisms is only in its infancy. Therefore, the aim of this study was to evaluate to what extent food intake, acute starvation and alterations in tumour polyamines modulate tumour growth by acute alterations in the energy state of the tumour.

Correspondence: K. Lundholm, Department of Surgery, Sahlgrenska Hospital, S-413 45 Göteborg, Sweden.
Received 10 December 1992; and in revised form 10 May 1993.

### Material and methods

### *Animal model*

Sex matched male and female mice (C57BI/6J) were used. All experiments were performed in 3 months old, weight-stable mice (20–25 g). A low- to undifferentiated tumour (MCG 101), originally induced as a sarcoma by methylcholanthrene, was used as a transplantable syngeneic tumour graft. The tumour tissue was implanted subcutaneously in the flank by a trocar. This tumour does not metastasise when implanted subcutaneously and its influence on the host has been reported elsewhere (Edström *et al.*, 1985; Lundholm *et al.*, 1978; Lundholm *et al.*, 1980). Whole body energy expenditure is increased in these tumour-bearing mice accounting for decreased food intake, but body temperature is normal (Lindmark *et al.*, 1983). Histological evaluation of tumour tissue, judged as being 'non-necrotic', has demonstrated a rather homogeneous capillary supply without areas of dead and lysed cells. The survival time of the tumour-bearing animals housed at + 25°C is 15–17 days and the cause of death has been evaluated elsewhere (Svaninger *et al.*, 1989).

The animals were kept in individual cages. They had free access to tap water and purina chow (Ewos, Astra, Södertälje, Sweden), deficient in polyamines and with metabolisable energy of 13.0 MJ $kg^{-1}$ = 3.1 Mcal $kg^{-1}$. Food intake (Lundholm *et al.*, 1980), changes in host body composition (Edén *et al.*, 1983) and tumour-growth rate (Karlberg *et al.*, 1981) have been reported. The animals were killed by cervical dislocation. In all experiments study and corresponding control animals were analysed at the same time and under identical conditions. Animals were lightly anaesthetised by i.p. injection of pentobarbital 0.06 mg $g^{-1}$ when necessary for the *in vivo* $^{31}$P-NMR experiments. This procedure did not influence tumour blood flow evaluated in separate experiments. Blood flow measurements were performed with a $^{132}$Xe clearance technique (Mattsson *et al.*, 1980).

### *Nuclear magnetic resonance spectroscopy ($^{31}$P-NMR)*

*In vivo* nuclear magnetic resonance spectroscopy was performed on a Bruker Biospec, BMT 24/30 at 2.35T giving an operating frequency of 40.55 MHz for phosphorus nuclei. The homogeneity of the magnetic field was optimised for each individual tumour and was accepted when the half-width of the $^1H$ signal was <0.5 ppm for one FID. Spectra were obtained by accumulating 128 alternatively 256 FIDs with a repetition rate of 1 s and a radio frequency pulse length of 75 μs (60° flip angle). Correction for the phosphorus resonances was made by the use of a pulse repetition rate of 16 s. The anaesthetised mice were placed with the tumour in a Helmholtz coil, 2 cm diameter. The relative concentrations of phosphocreatine (PCr), inorganic phosphate (Pi), phosphomonoesters (PME), phosphodiesters (PDE) and nucleotide triphosphate (NTP) were estimated from the computer integrated peak areas divided by the total phosphorus area (i.e. PCr + Pi + PME + PDE + α, β, γ-NTP) (Soussi *et al.*, 1990). The β-NTP resonance has the largest contribution from the β-adenosine triphosphate (β-ATP), (≈70%), it was therefore taken as a representative of the relative estimate of β-ATP content in the tissue and is referred to as ATP. The intracellular pH was determined from the chemical shift (δ) of the Pi relative to the PCr using the following equation: $pH = 6.75 + \log [(\delta\text{-}3.27)/(5.69\text{-}\delta)]$, (Gadian *et al.*, 1983). Initial experiments under different nutritional conditions using both *in vivo* (NMR) and *in vitro* (HPLC) techniques confirmed that the $^{31}P$-NMR signal represented tumour tissue metabolism and that possible signals from other host tissues could be neglected even in small tumours. Thus, a PCr/ATP ratio of 1.5 ± 0.3 (HPLC) and 1.6 ± 0.6, (NMR) was calculated for tumour tissue. For skeletal muscle the PCr/ATP ratio was 3.1 ± 0.8; $P < 0.01$.

*High performance liquid chromatography (HPLC)*

Non necrotic parts of tumour tissue were rapidly obtained by surgical dissection and frozen in liquid nitrogen and freeze-dried for 8 h (LYOVAC GT 2, Leybold-Heraeus). Tumour tissue was then minced to a powder. To 15 mg dry powder 0.285 ml PCA (1.5 mM) containing 1 M EDTA was added and the extraction was performed by gentle agitation for 20 min on ice. The precipitate was separated by centrifugation and neutralised before injection into the HPLC (Pharmacia Fine Chemicals AB, Uppsala, Sweden) (Idström *et al.*, 1990). The column used was a prepacked reversed phase C2/C18 Silca Column, Mino RPC S5/20 (5 μm; 4.6 × 200 mm), (Pharmacia). The eluation medium mobile phase consisted of 0.1 M ammonium dihydrogen phosphate buffer ($NH_4H_2PO_4$) with pH adjusted to 6.0 with 3 M ammonium hydroxide. The nucleotides, nucleosides, and purine bases, adenosinetriphosphate (ATP), adenosine diphosphate (ADP), adenosine monophosphate (AMP), inosine monophosphate (IMP), inosine (In), hypoxantine (Hx) and uric acid (Ua) as well as phosphocreatine (PCr) and creatine (Cr), were separated and concentrations were determined as described in details elsewhere (Soussi *et al.*, 1990). The energy charge was calculated according to Atkinson (Atkinson, 1977):

$$EC = ([ATP] + 1/2[ADP])/([ATP] + [ADP] + [AMP])$$

*Incorporation of bromodeoxyuridine (BrdUrd)*

The method for BrdUrd incorporation into tumour DNA is described in detail elsewhere (Van Furth & Van Zwet, 1988; Sasaki *et al.*, 1986; Gratzner, 1982). Briefly, the incorporation of BrdUrd was accomplished by an i.p. injection of $0.1\ mg\ g^{-1}$ bw BrdUrd dissolved in 0.9% NaCl, one hour before sacrifice (Wilson *et al.*, 1985). Tumour tissue was rapidly excised after sacrifice and washed with phosphate buffered saline (PBS). A single cell suspension was accomplished by cutting the tumour tissue into small pieces with a scalpel and then using collagenase ($1\ mg\ ml^{-1}$) in buffer solution. The mixture was then stirred with a magnetic follower for 10 min. The cell viability was around 95% as controlled by the trypan blue exclusion test before further analysis (Berry, 1974). The cell suspension was then centrifuged on to a slide using a cytocentrifuge an adequately dense monolayer of cells. Final preparation was then performed and immunoperoxidase-conjugated anti-BrdUrd exposed by diaminobenzidine (DAB) added and counterstained with Mayer. The percentage labelled cells indicated by BrdUrd incorporation into DNA was calculated from a total of 500 counted cells on each slide.

*Experiment 1: Energy state in tumour tissue during tumour progression*

In this experiment the animals were investigated on day 7, 9, 13 and 15. A spectrum of the tumour was obtained by $^{31}P$-NMR spectroscopy for *in vivo* analysis of the tumour energy state (Glickson *et al.*, 1987; Koutcher & Tyler 1984; Ng *et al.*, 1982; Rosen & Brady, 1983; Wehrle & Glickson, 1986). The tumour became palpable on day 5–6 and weighed around 0.5 g at this time point. Complete growth curves of the tumour have been published elsewhere (Karlberg *et al.*, 1981).

*Experiment 2: Energy state in tumour tissue following starvation and refeeding*

Animals were fed *ad libitum* until day 9 following tumour implantation. They were then starved with free access to water for 24 h followed by a 10 h refeeding period. The animals were anaesthetised and analyses were performed immediately before starvation 'freely-fed', after starvation 'starved' and after refeeding 'refed'. This protocol was used for both *in vivo* ($^{31}P$-NMR) and *in vitro* (HPLC) studies on the tumour bioenergetic status. Tumour tissue samples were also taken for measurement of Brd Urd incorporation into DNA.

*Experiment 3: Energy state in tumour tissue after alpha-difluoromethylornithine (DFMO) administration*

DFMO (MDL 71.782), a specific inhibitor of the enzyme ornithinedecarboxylase (ODC) (Pegg, 1988; Mamon *et al.*, 1976), was kindly supplied by Merrel-Dow Research Institute, Strasbourg, France. This compound was diluted in tap water (2%) and given continuously to the animals in drinking water from day 3 until day 9 when the animals were anaesthetised and studied *in vivo* ($^{31}P$-NMR). Mice consumed 4–5 ml of water per mouse per day. A parallel group of animals were killed following the NMR experiments for *in vitro* determinations (HPLC) of the tumour energy state.

*Statistics*

Differences between two groups were compared by the Student's *t*-test. One factor factorial ANOVA with or without repeated measures was used for multiple group comparisons. Results are given as mean ± SE; a 95% confidence interval was used in ANOVA computations; $P < 0.05$ is considered as statistically significant.

### Results

*Nutritional alterations*

Twenty-four hours starvation reduced animal weight from 22.2 ± 0.4 g to 18.0 ± 0.4 g. Subsequent refeeding for 10 h resulted in weight gain to 19.8 ± 0.4 g. The mean tumour weight during starvation/refeeding was 1.3 ± 0.2 g, 1.2 ± 0.2 g and 1.7 ± 0.1 g ($P < 0.05$) respectively recorded in 'Experiment 2', indicating that tumour growth was arrested during the starvation period and was then reinitiated during refeeding as reported earlier (Westin *et al.*, 1991*a*; Westin *et al.*, 1991*b*).

DFMO treatment (Experiment 3) by addition of 2% difluoromethylornithine to the drinking water caused a significant decrease in tumour weight, which was 1.5 ± 0.1 g in non-treated tumour animals compared to 1.0 ± 0.2 g in

DFMO treated animals ($P<0.05$). There was, no significant difference in carcass weight (22.3 ± 0.5 g and 22.1 ± 0.6 g) between the two groups. There was no influence on water and food intake by DFMO. The tumour concentration of putrescine and spermidine decreased, while spermine concentration was unchanged by DFMO as reported elsewhere (Westin et al., 1991b).

### *Tumour energy state*

The energy state of the tumour was assessed by $^{31}P$-NMR (*in vivo*) during tumour growth (Experiment 1), (Table I and Figure 2). The tumour was analysed with a size of 0.5–1 g on day 7 until a size of 4–6 g on day 15 just prior to animal death due to the tumour. During tumour progression the ATP/Pi and PCr/Pi were slightly reduced while the PME and the PDE levels were increased (Figure 2). There was no significant change in the tumour pH during tumour progression. Table II contains data from *in vivo* ($^{31}P$-NMR) determinations and Table III contains *in vitro* (HPLC) determinations of energy rich phosphates in tumour tissue following 24 h starvation and subsequent 10 h refeeding and following DFMO treatment of tumour-bearing animals. The energy charge in tumour tissue decreased significantly during starvation and returned to normal tumour levels during refeeding. The PCr/Pi and the ATP/Pi were reduced, although the reductions did not reach statistical significance during starvation. Subsequent refeeding resulted in a significant overshoot of these phosphates compared to levels in tumour tissue from freely-fed animals. Inorganic phosphate decreased during refeeding. *In vivo* $^{31}P$-NMR and *in vitro* determinations gave qualitatively comparable results (Table II *vs* III). Thus, light pentobarbital anaesthesia had no effect on tumour content of high energy phosphates, (the *in vitro* determination of phosphates was done without prior use of anaesthesia). Starvation/refeeding was associated with alterations in tumour blood flow (Figure 1).

**Figure 1** Tumour blood flow in animals that were freely fed, then subjected to a period of 10 h complete starvation, and then refed again for 4 h. The decline in blood flow was significant at $P<0.05$. Blood flow was measured as described in Material and methods.

**Table I** Energetic parameters in tumour tissue measured by *in vivo* $^{31}P$-NMR during tumour progression. Measurements were repeated on day 7–15 in the same animals ($n$ = 6); values are mean ± s.e. The tumour weight was 0.5–1 g day 7 and 4–6 g day 15

| *Day* | *7* | *9* | *13* | *15* | *ANOVA P<* |
|---|---|---|---|---|---|
| PME | 3.7 ± 0.2 | 6.0 ± 0.6 | 4.5 ± 0.4 | 14.4 ± 3.8 | 0.05 |
| Pi | 9.6 ± 0.1 | 13.1 ± 1.0 | 9.5 ± 0.7 | 25.9 ± 7.2 | 0.05 |
| PDE | 9.1 ± 0.9 | 18.7 ± 1.6 | 9.6 ± 1.7 | 14.2 ± 1.4 | 0.05 |
| PCr | 25.3 ± 1.0 | 16.0 ± 2.1 | 18.4 ± 0.5 | 10.0 ± 0.6 | 0.01 |
| ATP | 9.5 ± 1.0 | 7.4 ± 1.2 | 8.0 ± 0.2 | 5.9 ± 1.6 | |
| pH | 7.3 ± 0.02 | 7.1 ± 0.04 | 7.2 ± 0.04 | 7.2 ± 0.01 | |
| PCr/Pi | 2.6 ± 0.5 | 1.4 ± 0.3 | 2.0 ± 0.1 | 0.6 ± 0.1 | 0.01 |
| ATP/Pi | 1.0 ± 0.1 | 0.5 ± 0.1 | 0.9 ± 0.1 | 0.4 ± 0.1 | 0.01 |

One factor ANOVA for repeated measures was used for the statistical evaluation. Metabolite levels are in per cent of total phosphorus content as described in Materials and methods.

**Table II** Energetic parameters assessed *in vivo* on day 9 by $^{31}P$-NMR in tumour-bearing (TB) mice that were either freely-fed ($n$ = 9), starved for 24 h ($n$ = 10) and subsequently refed for 10 h ($n$ = 5) or freely-fed DFMO-treated ($n$ = 5); values are mean ± s.e.

| | *Freely-fed TB-animal* | *Starved TB-animal* | *Refed TB-animal* | *Freely-fed TB-animals receiving DFMO* | *ANOVA P<* |
|---|---|---|---|---|---|
| PME | 4.2 ± 0.8 | 6.7 ± 2.6 | 1.3 ± 0.7 | 2.6 ± 1.4 | |
| Pi | 8.4 ± 1.8 | 11.0 ± 2.9[b] | 1.3 ± 0.3 | 3.4 ± 0.4 | 0.05 |
| PDE | 12.4 ± 2.5 | 9.9 ± 1.5 | 5.2 ± 1.7[a] | 6.0 ± 1.2 | |
| PCr | 19.3 ± 1.2 | 18.3 ± 2.6 | 22.3 ± 1.7 | 30.2 ± 6.7[a] | 0.05 |
| ATP | 11.3 ± 2.9 | 13.5 ± 2.2 | 16.1 ± 2.3 | 19.0 ± 1.3 | 0.08 |
| pH | 7.1 ± 0.1 | 6.9 ± 0.2 | 7.2 ± 0.1 | 7.2 ± 0.1 | |
| PCr/Pi | 5.8 ± 2.4 | 3.4 ± 1.1[b] | 23.7 ± 6.3[a] | 9.4 ± 2.1 | 0.0004 |
| ATP/Pi | 5.7 ± 3.0 | 2.6 ± 0.9[b] | 16.9 ± 4.9[a] | 6.3 ± 1.4 | 0.008 |

One factor factorial ANOVA was used for the statistical evaluation. [a]Significantly different ($P<0.05$) *vs* freely-fed and starved TB-animals. [b]Significantly different ($P<0.05$) *vs* refed TB-animals.

**Table III** HPLC analysis of phosphocreatine, creatine, nucleotides, nucleosides and purine bases in tumour biopsy specimens from either tumour-bearing (TB) mice (on day 9) that were freely-fed ($n = 8$), starved for 24 h ($n = 6$) subsequently refed for 10 h ($n = 6$) or freely-fed DFMO-treated ($n = 5$). The results can be compared to *in vivo* measurements in Table II, which were done before the animals were killed. Values are mean ± s.e. and are expressed as μmol $g^{-1}$ dry weight

| | *Freely-fed TB-animal* | *Starved TB-animal* | *Refed TB-animal* | *Freely-fed TB-animals receiving DFMO* | *ANOVA P <* |
|---|---|---|---|---|---|
| Energy charge | 0.61 ± 0.02 | 0.52 ± 0.03[a] | 0.69 ± 0.02[a,b] | 0.65 ± 0.02 | 0.0008 |
| ATP | 4.6 ± 0.5 | 4.3 ± 0.6 | 7.3 ± 0.8[a,b] | 7.0 ± 1.6 | 0.007 |
| ADP | 3.4 ± 0.3 | 4.2 ± 0.3[a] | 4.1 ± 0.2[a] | 4.4 ± 0.3 | 0.05 |
| AMP | 2.2 ± 0.1 | 3.5 ± 0.3[a] | 2.0 ± 0.2[b] | 2.7 ± 0.5 | 0.001 |
| IMP | 2.0 ± 0.1 | 2.9 ± 0.1[a] | 1.5 ± 0.3[b] | 2.4 ± 0.2 | 0.0002 |
| Inosine | 3.5 ± 0.3 | 3.8 ± 0.2 | 3.8 ± 0.2 | 4.1 ± 0.2 | |
| Hypoxanthine | 1.3 ± 0.1 | 1.6 ± 0.2 | 2.5 ± 0.1[a,b] | 1.4 ± 0.4 | 0.0001 |
| Uric acid | 2.7 ± 0.2 | 2.2 ± 0.4 | 1.4 ± 0.2[a,b] | 1.4 ± 0.4 | 0.003 |
| PCr | 5.9 ± 0.6 | 4.8 ± 0.6 | 4.0 ± 0.3[a] | 11.9 ± 1.4 | 0.06 |
| Cr | 36.6 ± 3.4 | 36.3 ± 6.1 | 37.1 ± 2.4 | 49.1 ± 2.6 | 0.03 |

One factor factorial ANOVA was used for the statistical evaluation. [a]Significantly different ($P < 0.05$) *vs* freely-fed TB-animals. [b]Significantly different ($P < 0.05$) *vs* starved TB-animals.

**Figure 2** Time course of relative changes in PCr/Pi (□), ATP/Pi (○) and in PME (▲), in tumour tissue determined by *in vivo* $^{31}$P-NMR spectroscopy in the same animals following tumour implantation on day 0. The tumours become palpable and anorexia starts on day 5–7 in tumour-bearing mice. Most animals die spontaneously on day 17. Bars represent s.e. ($n = 6$).

**Figure 3** Relative change in ATP concentration in tumour tissue during starvation – refeeding and following DFMO-treatment in relation to DNA-synthesis in the tumour. Animals were starved and refed or treated with DFMO (difluoro-methylornithine) as described in Material and methods. Bars represent SE, $n = 10$ in each group. The ATP was determined by HPLC (Table III). DNA-synthesis was estimated by bromodeoxy-uridine (BrdUrd) incorporation into tumour cell DNA. *$P < 0.05$ *vs ad lib* (freely-fed tumour-bearing animals).

DFMO treatment (Experiment 3) caused a significant rise in the high energy phosphates (PCr, $P < 0.05$ and ATP, $P < 0.08$) measured both *in vivo* and *in vitro*. There was no significant difference in either pH or energy charge (0.63 ± 0.06) in DFMO-treated tumour-bearing groups compared to untreated control TB-animals (Tables II and III).

### *Tumour DNA synthesis*

Acute starvation decreased BrdUrd incorporation into DNA by 20% ($P < 0.01$) and this attenuation was restored during subsequent refeeding. DFMO treatment decreased DNA-synthesis in tumour tissue (Figure 3).

### Discussion

Previous experiments in our tumour model have demonstrated that the carbohydrate component in a standard chow diet initiates tumour DNA synthesis during refeeding which was probably not related to insulin (Westin *et al.*, 1991*a*). The aim of this study was, therefore, to evaluate whether food intake and acute starvation modulate tumour growth and DNA synthesis indirectly by rapid alterations in the energy state of the tumour, since ATP and ADP are important elements in allosteric regulation of glycolysis and respiration. Polyamine synthesis inhibition was used as an alternative approach to decrease tumour DNA synthesis and thereby indirectly energy rich phosphates.

Our results demonstrate that the energy charge was significantly decreased in tumours on starved animals, while it rapidly returned to pre-starvation levels during refeeding. These findings confirm that the metabolism in rapidly growing experimental tumours are sensitive to acute changes in the host nutrition state. A nutrition related growth-phenomenon in experimental tumours has also been illustrated indirectly by giving tumour-bearing rats intravenous nutrition, which resulted in a more rapid tumour growth compared to orally nourished tumour-bearing animals with anorexia (Torosian, 1984). By this mechanism anorexia may be a counter-regulatory phenomenon to slow down tumour growth and thereby maximise survival. The present study suggests that nutrition-induced alterations in tumour growth are in part explained by alterations in tumour content of energy phosphates, probably related to a reduced tumour blood flow.

We have recently demonstrated that a decrease in DNA synthesis following starvation was preceded by a similar change in tumour ornithine-decarboxylase activity (ODC), (Westin *et al.*, 1991*b*). ODC-activity can be irreversibly inhibited by DFMO (difluoro-methyl-ornithine), a substance which is under clinical evaluation for tumour treatment. In present experiments DFMO inhibited tumour growth as expected (Pegg, 1988), as confirmed by a lower BrdUrd incorporation into DNA and a 35% decrease in tumour mass. It is important to emphasise that food intake was not decreased in DFMO-treated tumour-bearing animals. Therefore, we conclude that DFMO-inhibition of tumour growth was direct rather than indirect by altered nutrition and decreased availability of energy phosphates. In line with this theory, both ATP and PCr levels were increased and Pi decreased in tumour tissue during DFMO-inhibition of tumour growth. Therefore, we did not measure tumour blood flow in DFMO treated animals, since they did not show a decline in tumour high energy phosphates.

The energy charge in tumour tissue was under all conditions significantly lower than 0.85, which suggests a different equilibrium for energy production/utilisation in tumour tissue than seen in normal tissues (Atkinson, 1977). This is not to say that the metabolic rate was high in our tumours, but may rather represent a different equilibrium for energy flow compared to normal cells. We have previously confirmed that respiration and glycolysis is lower in these tumour cells than found in many normal cells (unpublished results). The overall tumour concentration of the high-energy phophates was also lower than seen in most normal tissues. This may to some extent depend on the fact that tumour tissue is composed of a mixture of cells with high and low viability. Another explanation may be that tumour profusion is heterogeneous and that tumour blood flow was considerably lower than found in normal tissues as liver and skeletal muscles (unpublished). Blood flow is the major determinant of substrate and oxygen supply to the tissue. Insufficient tumour perfusion implicates poor substrate supply and low oxygen concentration which probably affects the tumour by reducing its energy state. Therapy induced decreases in the nucleotide triphosphates-to-Pi ratio have been attributed to decreased blood flow (Evelhoch et al., 1988). However, necrosis will occur only when the ATP is depleted to monophosphates and further irreversibly degraded to nonphosphorylated products. In a study investigating the effects of the cytokine interleukin-α on RIF-1 tumours, necrosis appeared at least 12 h after an important reduction in phosphate metabolites (Constantinidis et al., 1989).

Our findings of high energy phosphates in tumour tissue agree with previous results obtained by NMR spectroscopy on human (Daly & Cohen, 1989; Vaupel et al., 1989) and experimental tumours (Smith et al., 1989). Major increases in Pi compared with the other phosphate resonances have been shown to be a result of tumour necrosis (Naruese et al., 1985). The PME levels found to increase with tumour progression support the usefulness of this $^{31}$P NMR signal in monitoring tumour energetics. However, any AMP, IMP and sugar-phosphates formed would also contribute to the PME peak which contains contributions from phosphoethanolamine and phosphocholine. It should be pointed out that a direct comparison between the HPLC and the NMR data could be misleading since analysis of tissue extracts reflects total metabolite concentrations and not free cytosolic contents as by *in vivo* NMR. The PCr instability and its rapid breakdown to Pi and Cr is not avoidable in biochemical analysis. However, assessing this metabolite *in vivo* by NMR may correct for the usually lower extract values.

In conclusion, this study gives support to a direct role of ODC for induction or promotion of tumour cell division. A decrease in ODC-activity by DFMO (Pegg, 1988) led to inhibition of DNA-synthesis, but this decrease was not explained by a decreased availability of cellular energy. On the contrary, a lowered DNA synthesis following ODC-inhibition by DFMO was associated with a rise in tumour energy rich phosphates probably secondary to a decreased energy demand for cell division. In contrast, a fall in DNA synthesis following starvation led to a decrease in tumour energy content perhaps related to a lower blood flow. Our findings give a rationale to suggest that force-feeding of energy-deprived tumours may stimulate tumour growth provided appropriate tumour oxygenation (Vaupel et al., 1989). In this respect carbohydrate containing diets may be most detrimental (Westin et al., 1991a). In other words, force-feeding by glucose may activate ODC, which may induce DNA synthesis and thereby promote cell cycle traverse. These events may then give a more susceptible situation for cytocidal drug therapy. This hypothesis can now be systematically tested in experimental and clinical tumour systems.

Supported in parts by grants from the Swedish Cancer Society (93-B89-22XA, 2014-B88-01XA, 2147-B89-04XA), the Medical Research Council (Project No. 536, 8712), Tore Nilson Foundation, Assar Gabrielsson Foundation (AB Volvo), Jubileumskliniken Foundation, The Harald & Greta Jeansson Foundation, Ingabritt & Arne Lundberg Research Foundation, Axel & Margaret Ax:son Johnson Foundation, Swedish and Göteborg Medical Societies and the Medical Faculty, University of Göteborg.

### References

ATKINSON, D. (1977). *Cellular Energy Metabolism and its Regulation.* Academic Press, NY.

BERRY, M.N. (1974). High-yield preparation of morphologically intact isolated parenchyma cells from rat liver. *Meth. Enzym.*, **32**, 625–632.

CONSTANTINIDIS, I.C., BRAUNSCHWEIGER, P.G., WEHRLE, J.P., KUMAR, N., JOHNSON, C.S., FURMANSKI, P. & GLICKSON, J.D. (1989). $^{31}$P-Nuclear magnetic resonance studies of the effect of recombinant human interleukin 1 alpha on the bioenergetics of RIF-1 tumors. *Cancer Res.*, **49**, 6379–6382.

DALY, P.F. & COHEN, J.S. (1989). Magnetic resonance spectroscopy of tumors and potential *in vivo* clinical applications: a review. *Cancer Res.*, **49**, 770–779.

EDÉN, E., LINDMARK, L., KARLBERG, I. & LUNDHOLM, K. (1983). Role of whole body lipids and nitrogen as limiting factors for survival in tumor-bearing mice with anorexia and cachexia. *Cancer Res.*, **43**, 3707–3711.

EDSTRÖM, S., REINHOLDTSEN, L. & LUNDHOLM, K. (1985). Glucose turnover in adult non-growing tumour-bearing mice. *Int. J. Biochem.*, **17**, 649–652.

EVELHOCH, J.L., BISSERY, M.C., CHABOT, G.G., SIMPSON, N.E., MCCOY, C.L., HEILBRUN, L.K. & CORBETT, T.H. (1988). Flavone acetic acid (NSC 34 7512)-induced modulation of murine tumor physiology monitored by *in vivo* nuclear magnetic resonance spectroscopy. *Cancer Res.*, **48**, 4749–4755.

GADIAN, D.G., RADDA, G.K., DAWSON, M.J. & WILKIE, D.R. (1982). PHi measurements of cardiac and skeletal muscle using $^{31}$P NMR: Intracellular PH: its measurements, regulation and utilization in cellular functions. A.R. Liss Inc, NY. 61–77.

GLICKSON, J., EVANOCHKO, W., SAKAI, T. & NG, T. (1987). *In vivo* NMR spectroscopy of tumors. In Gupta, R.K. (ed.) *NMR Spectroscopy of Cells and Organisms.* Boca Raton FL: CEC Press Inc. 1, 99–134.

GRATZNER, H.G. (1982). Monoclonal antibody to 5-Bromo- and 5-Iododeoxyridine: A new reagent for detection of DNA replication. *Science*, **218**, 474–475.

IDSTRÖM, J.P., SOUSSI, B., WANAG, E. & BYLUND-FELLENIUS, A.C. (1990). Analysis of purine nucleotides in muscle tissue by HPLC. *Scand. J. Clin. Lab. Invest.*, **50**, 541–549.

JÄNNE, J., PÖSÖ, H. & RAINA, A. (1978). Polyamines in rapid growth and cancer. *Biochim Biophys Acta.*, **473**, 241–293.

KARLBERG, I., EDSTRÖM, S., EKMAN, L., JOHANSSON, S., SCHERSTÉN, T. & LUNDHOLM, K. (1981). The metabolic host-reaction in response to the proliferation of non-malignant cells versus that of malignant cells *in vivo*. *Cancer Res.*, **41**, 4154–4161.

KOUTCHER, J. & TYLER, B. (1984). Principles of nuclear magnetic resonance. *J. Nucl. Med.*, **25**, 101–111.

LINDMARK, L., EDSTRÖM, S., EKMAN, L., KARLBERG, I. & LUNDHOLM, K. (1983). Energy metabolism in non-growing mice with sarcoma. *Cancer Res.*, **43**, 3649–3654.

LUNDHOLM, K., EDSTRÖM, S., EKMAN, L., KARLBERG, I., BYLUND, A.-C. & SCHERSTÉN, T. (1978). A comparative study of the influence of malignant tumor on host metabolism in mice and man. Evaluation of an experimental model. *Cancer*, **42**, 453–461.

LUNDHOLM, K., EDSTRÖM, S. KARLBERG, I., EKMAN, L. & SCHERSTÉN, T. (1980). Relationship of food intake, body composition, and tumor growth to host metabolism in non-growing mice with sarcoma. *Cancer Res.*, **40**, 2516–2522.

MAMONT, P.S., BÖHLEN, P., MCCANN, P.P., BEY, P., SCHUBER, F. & TARDIF, C. (1976). Alpha-methyl-ornithine, a potent competitive inhibitor of ornithine decarboxylase, blocks proliferation of rat hepatoma cells in culture. *Proc. Natl Acad. Sci. USA.*, **73**, 1626–1630.

MATTSSON, J., ALPSTEN, M., KARLSSON, L. & PETERSON, H.I. (1980). Influence of noradrenaline on local tumour blood flow. *Eur. J. Cancer*, **16**, 99–102.

NARUESE, S., HIRAKAWA, K., HORIKAWA, Y., TANAKA, C., HIGUCHI, T., UEDA, S., HISHIKAWA, H. & WATARI, H. (1985). Measurements of *in vivo* $^{31}$P nuclear magnetic resonance spectra in neuroectodermal tumors for the evaluation of the effects of chemotherapy. *Cancer Res.*, **45**, 2429–2433.

NG, T., EVANOCHKO, W., HIRAMOTO, R., GHANTA, V., LILLY, M., LAWSON, A., CORBETT, T., DURANT, J. & GLICKSON, J. (1982). 31P NMR spectroscopy of *in vivo* tumors. *J. Magnetic Resonance*, **49**, 271–286.

OTA, D.M., NISHIOKA, K., GROSSIE, V.B. & FOULKES, M. (1984). Nutritional parameters affecting erythrocyte polyamine levels in cancer patients. *J. Clin. Oncol.*, **2**, 1157–1164.

PEGG, A.E. (1988). Polyamine metabolism and its importance in neoplastic growth and as a target for chemotherapy. *Cancer Res.*, **48**, 759–774.

POPP, M.B., MORRISON, S.D. & BRENNAN, M.F. (1981). Total parenteral nutrition in a methylcholanthrene-induced rat sarcoma model. *Cancer Treat Rep.*, **65**, 137–143.

ROSEN, B. & BRADY, T. (1983). Principles of nuclear magnetic resonance for medical application. *Seminars in Nuclear Medicin*, no 4. **13**, 308–318.

RUSSELL, D.H. (1973). *Polyamines in Growth – Normal and Neoplastic.* Raven Press: New York.

SASAKI, K., MURAKAMI, T., OGINO, T., TAKAHASHI, M. & KAWASAKI, S. (1986). Flow cytometric estimation of cell cycle parameters using a monoclonal antibody to bromodeoxyuridine. *Cytometry*, **7**, 391–395.

SAUER, L.A., NAGEL, W.O., DAUCHY, R.T., MICELI, L.A. & AUSTIN, J.E. (1986). Stimulation of tumor growth in adult rats *in vivo* during an acute fast. *Cancer Res.*, **46**, 3469–3475.

SMITH, S.R., MARTIN, P.A., DAVIES, J.M. & EDWARDS, R.H.T. (1989). Characterization of the spleen by *in vivo* image guided $^{31}$P magnetic resonance spectroscopy. *NMR in Biomed.*, **24**, 172–178.

SOUSSI, B., IDSTRÖM, J.P., BYLUND-FELLENIUS, A.C. & SCHERSTÉN, T. (1990). Dynamics of skeletal muscle energetics during ischemia and reperfusion assessed by *in vivo* $^{31}$P NMR. *NMR in Biomed.*, **3**, 71–77.

STRAGAND, J., BRAUNSCHWEIGHER, P.G., POLLICE, A.A. & SCHIFFER, L.M. (1978). Cell kinetic alterations in murine mammary tumors following fasting and refeeding. *Eur. J. Cancer*, **15**, 281–286.

SVANINGER, G., GELIN, J. & LUNDHOLM, K.G. (1989). The cause of death in non-metastasizing sarcoma-bearing mice. A study with relevance for tumor treatment experiments in mice. *Eur. J. Cancer Clin Oncol.*, **25**, 1295–1302.

TOROSIAN, M.H., TSOU, K.C., DALY, J.M., MULLEN, J.L., STEIN, T.P., MILLER, E.E. & BUZBY, G.P. (1984). Alteration of tumor cell kinetics by pulse total parenteral nutrition. *Cancer*, **53**, 1409–1415.

VAN FURTH, R. & VAN ZWET, T. (1988). Immunocytochemical detection of 5-bromo-2-deoxyuridine incorporation in individual cells. *J. Immunol. Methods*, **108**, 45–51.

VAUPEL, P., OKUNIEFF, P., KALLINOWSKI, F. & NEURINGER, L.J. (1989). Correlations between $^{31}$P-NMR spectroscopy and tissue $O_2$ tension measurements in a murine fibrosarcoma. *Radiation Res.*, **120**, 477–493.

WEHRLE, J. & GLICKSON, J. (1986). $^{31}$P NMR spectroscopy of tumors *in vivo*. *Cancer Biochem. Biophys.*, **8**, 157–166.

WESTIN, T., EDSTRÖM, S. & LUNDHOLM, K.G. (1991*a*). Ornithine decarboxylase activity in tumor tissue in response to refeeding and its dependency on diet components. *Eur. J. Cancer*, **27**, 1282–1288.

WESTIN, T., GUSTAVSSON, B. EDSTRÖM, S., HELLANDER, K., REINHOLDTSEN, L., TIBELL, L. & LUNDHOLM, K. (1991*b*). Tumor cytokinetic effects of acute starvation versus polyamine depletion in tumor-bearing mice. *Cytometry*, **12**, 628–635.

WILSON, S., MCNALLY, N., DUMPHY, E., KÄRCHER, H. & PFRAGNER, R. (1985). The labelling index of human and mouse tumors assessed by bromodeoxyuridine staining *in vitro* and *in vivo* and flow cytometry. *Cytometry*, **6**, 641–647.